# Investigation on Comparison of Morphological Characteristics of Various Coarse Aggregates before and after Abrasion Test

**DOI:** 10.3390/ma13020492

**Published:** 2020-01-20

**Authors:** Saisai Zhang, Jianzhong Pei, Rui Li, Yong Wen, Jiupeng Zhang

**Affiliations:** School of Highway, Chang’an University, Xi’an 710064, China; zhangssai@126.com (S.Z.); lirui@chd.edu.cn (R.L.); wenyong@chd.edu.cn (Y.W.); zhjiupeng@chd.edu.cn (J.Z.)

**Keywords:** coarse aggregates, mineral composition, Los Angeles abrasion test, morphological characteristics, Aggregate Image Measurement System

## Abstract

Under the repeated loading, the continuous impact and friction of tires on aggregates resulted in some changes in their morphology, which may cause rutting, decrease in skid resistance, and fatigue damage of the road. In order to explore specific changes in coarse aggregate morphology, the Los Angeles abrasion test was used to simulate the force exerted on coarse aggregates and the morphologies of different aggregates before and after abrasion were compared. Four types of coarse aggregates were selected and their mineral compositions were analyzed by X-Ray Diffraction (XRD). The morphological characteristics were measured using Aggregate Image Measurement System (AIMS-Ⅱ), including angularity, surface texture, sphericity and Flat and Elongation (F and E) ratio. Results showed that the angularity value for each type of aggregates significantly reduced after abrasion and the angularity reductions of various aggregates were consistent with the results of abrasion test, indicting the angularity reduction was the main component of abrasion loss. Whereas, there was no significant different between the surface texture of coarse aggregates before and after abrasion. For shape properties, both sphericity and F and E ratio results showed that aggregates with excessively high F and E ratio were easy to break, which might cause rutting and were harmful to pavement. Therefore, for pavements with high performance requirement, coarse aggregates with large angularity and low abrasion value should be preferred, whereas the quantity of particles with excessively high F and E ratio should be controlled.

## 1. Introduction

Under the repeated traffic loading, various damages such as rutting, decrease in skid resistance, water damage and fatigue cracking occurred on the pavement. Many previous studies showed that these pavement diseases are closely related to the properties of coarse aggregates [1,2,3]. In wearing course, the abrasion resistance of aggregates has a strong correlation with the skid resistance of pavement [4]. Liang and Chyi investigated the polishing behavior of aggregates and found the abrasion resistance of aggregates provided the main skid resistance for pavement [5]. And along with physical properties (i.e., water absorption, abrasion and impact) [6,7,8,9], the morphological characteristics of coarse aggregates (i.e., shape, angularity, and surface texture) have a direct correlation with the functional and structural performance of pavement layers [10,11,12,13]. Several researchers have reported coarse aggregates with good morphology can provide enough strength and good structural stability to the asphalt pavement [14,15,16]. Beside the crushed stone aggregate, the recycled aggregates’ abrasion resistance and morphological characteristics also have a significant effect on the pavement performances of asphalt mixture [17,18,19].

Shape represents the profile boundary of aggregates and directly affects the fatigue resistance of asphalt mixtures [20,21]. Cubic aggregates can be embedded in each other to form a stable skeleton structure, whereas flat or elongated aggregates are easy to break under the repeated load of vehicles [22]. The large Flat and Elongation (F and E) ratio resulted in aggregate breakage and degradation under repeated loads [23]. Angularity shows the corner sharpness of aggregates, which has a significant influence on the anti-skid performance and rut resistance of asphalt pavement [24,25,26]. Shah and Abdullah designed three different mixtures using the angular, elongated and flaky coarse aggregates separately [27]. They found the mixtures mixed with angular aggregates showed the best skid resistance whereas the flaky shape demonstrated the lowest skid resistance. Texture is the surface roughness of aggregates, which belongs to the microscopic scale of coarse aggregates. The rough surface is able to improve the bond strength between asphalt and aggregates, which is beneficial to the resistance to moisture damage of asphalt pavement [28,29]. Bessa investigated the effect of angularity and texture on the mechanical behavior of asphalt mixtures, and results showed asphalt mixes with aggregates having high angularity and surface texture were found to perform better for all mechanical tests [30]. It can be seen that there is a significant correlation between the pavement performance and coarse aggregate morphologies, so the changes in the morphological characteristics of coarse aggregates will directly affect performances of the pavement.

To explore the morphology changes occurred in the coarse aggregates and find the cause of pavement damage, the effect of abrasion test on morphologies of different coarse aggregates is to be investigated. The Los Angeles abrasion test will be used to simulate the similar force received by coarse aggregates in the pavement, which can reflect aggregates’ ability to resist impact and rub. Four types of coarse aggregates, including limestone, granite, diabase, and tuff, will be selected, and their mineral compositions will be analyzed by XRD. The morphological characteristics before and after the abrasion test are to be measured using AIMS system, including angularity, surface texture, sphericity, and F and E ratio. This study has practical guidance for predicting and improving the pavement performance of asphalt mixtures.

## 2. Experimental Parts

### 2.1. Materials and Instruments

Four types of coarse aggregates, including limestone, tuff, granite, and diabase, were collected from Shaanxi Province. Specifically, the limestone aggregates were collected from Weinan City, the tuff aggregates were collected from Shangluo City, the granite aggregates were collected from Xi’an City and the diabase aggregates were collected from Ankang City. These aggregates were sieved, washed and dried for further testing. For XRD test, the D/max-2500 X-ray diffractometer manufactured by Rigaku (Tokyo, Japan) was used and each type of aggregate was ground into a powder of less than 0.075 mm. For Los Angeles abrasion test, the YX-G abrasion tester made by Xi’an Yaxing Civil Instrument Co., Ltd (Xi’an, China) was used and coarse aggregates of 13.2–16 mm and 16–19 mm were prepared, and each size was 2.5 kg. For AIMS test, the AIMS-Ⅱ instrument manufactured by American Pine Instrument Company (Grove City, PA, USA) was used and 13.2–16 mm aggregates before and after the abrasion test were selected randomly to measure the morphological properties.

### 2.2. Los Angeles Abrasion Test

The Los Angeles abrasion test was conducted using the Los Angeles method in accordance with the Highway Engineering Aggregate Test Procedures (JTG42-2005). Specifically, mixed the coarse aggregates of 13.2–16 mm and 16–19 mm into the cylinder of the abrasion tester and each size was 2.5 kg. Added the steel balls to the cylinder, started the abrasion tester to rotate at a speed of 30 r/min~33 r/min and stopped it at the required number of revolutions. Then, sieved the sample using a square sieve of 1.7 mm and rinsed off the left aggregates. Lastly, dried it using the oven at 105 °C to constant weight and weighed. The ratio of the mass loss to initial mass is defined as abrasion value (Equation (1)).

(1) $$Q\left(\%\right)=\frac{m_{1}-m_{2}}{m_{1}}\times 100\%$$

where *Q* (%) is the abrasion value, *m_1_* index is initial mass of aggregates and *m_2_* index is the mass of aggregates after abrasion.

### 2.3. Aggregate Image Measurement System (AIMS) Test

**Figure 1:**
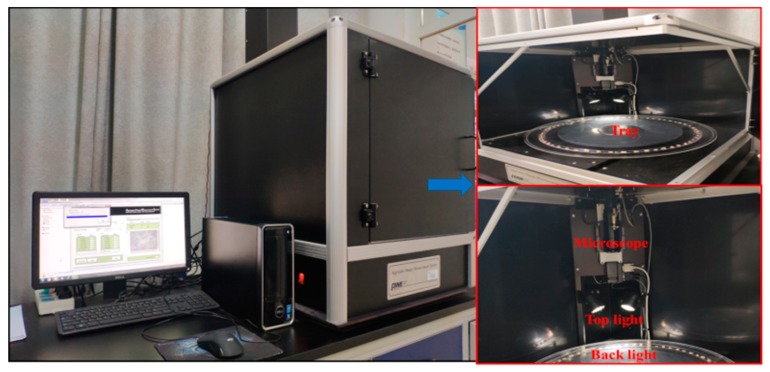
AIMS-Ⅱ instrument.

The AIMS enables accurate and repeatable measurement of morphological characteristics of aggregates [31]. It is a computer-based digital image system, which can measure both the morphologies of fine and coarse aggregates, ranging from 0.075 to 37.5 mm sieve [32]. This system consists of a microscope camera, aggregates tray and a back and top lighting system [33], which was shown in Figure 1. The angularity, sphericity, F and E ratio, and surface texture are scanned for coarse aggregates, whereas angularity and Form2D are analyzed for fine aggregates [34].

#### 2.3.1. Angularity

Angularity represents the corner sharpness of coarse aggregates, which is calculated by the gradient method. The gradient change along the particle boundary is calculated and the average change in gradient vector is regarded as the angularity, showing as Equation (2) [35]. The scale of angularity is from 0 to 10,000 and is classified in 4 different ranges. Low angularity represents the rounded particle, whereas high angularity represents the angular aggregate.

(2) $$\mathrm{Angularity}=\frac{1}{\frac{n}{3}-1}\overset{n-3}{\underset{i=1}{\sum }}\left|\theta_{i}-\theta_{i+3}\right|,$$

where *n* is the number of points, *i* is the i point on the edge of the particle and *θ* is the orientation angle of the edge points.

#### 2.3.2. Surface Texture

Surface texture is the surface roughness of coarse aggregate, which is determined by the grayscale images of aggregates surfaces. The wavelet decomposition is used in AIMS to determine the texture [36]. The scale of texture is from 0 to 1000 and is classified in 4 different ranges. The low texture value represents the smooth surface, whereas the high texture value represents the rough surface.

(3) $$\mathrm{Texture}=\frac{1}{3N}\overset{3}{\underset{i=1}{\sum }}\overset{N}{\underset{j=1}{\sum }}{\left[D_{i,j}\left(x,y\right)\right]}^{2},$$

where *N* is the coefficients number in an image, *i* is for detailed images, *j* is the wavelet index and *D* is the decomposition.

#### 2.3.3. Sphericity

Sphericity is a measure of the 3D shape properties for an aggregate, which is determined by three dimensions, namely the longest dimension, the shortest dimension, and the intermediate dimension. The scale of texture is from 0 to 1 and is classified in 4 different ranges. The higher the sphericity is, the better the shape of an aggregate is.

(4) $$\mathrm{Sphericity}=\sqrt[{3}]{\frac{d_{S}d_{I}}{d_{L}^{2}}},$$

where *d_L_* index is the longest dimension of the aggregate, *d_S_* index is the shortest dimension of the aggregate, and *d_I_* index is the intermediate dimension of the aggregate.

#### 2.3.4. Flat and Elongation (F and E) Ratio

F and E is the ratio of the longest dimension to the shortest dimension for an aggregate, which is shown as Equation (5). The flat or elongated aggregates are easy to break under the repeated load of vehicles, so the Superpave specification allows no more than 10% of coarse aggregates with F and E ratio greater than 5:1.

(5) $$F\;\mathrm{and}\;E=\frac{d_{L}}{d_{S}}$$

where the meaning of *d_L_* index and *d_S_* index is same to Equation (4).

## 3. Results and Discussion

### 3.1. Mineral Compositions

**Figure 2:**
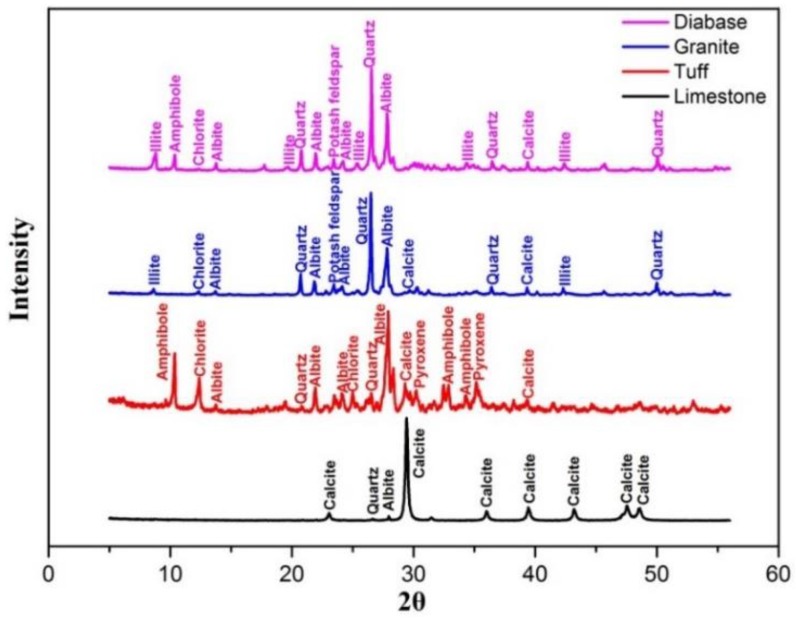
XRD patterns of four aggregates.

The XRD pattern of four aggregates was shown in Figure 2. It can be seen that the compositions of four aggregates were significantly different. The main component of the limestone was calcite, which accounted for the vast majority. The tuff in this study was mainly composed of albite, amphibole and chlorite. The granite was mainly composed of quartz and albite. The main component of diabase is quartz, albite and amphibole (some has been metamorphic to illite). Since the mineral composition of aggregates have a great influence on its properties, the morphology of different aggregates may change differently after abrasion test.

### 3.2. Abrasion Value Analysis

**Figure 3:**
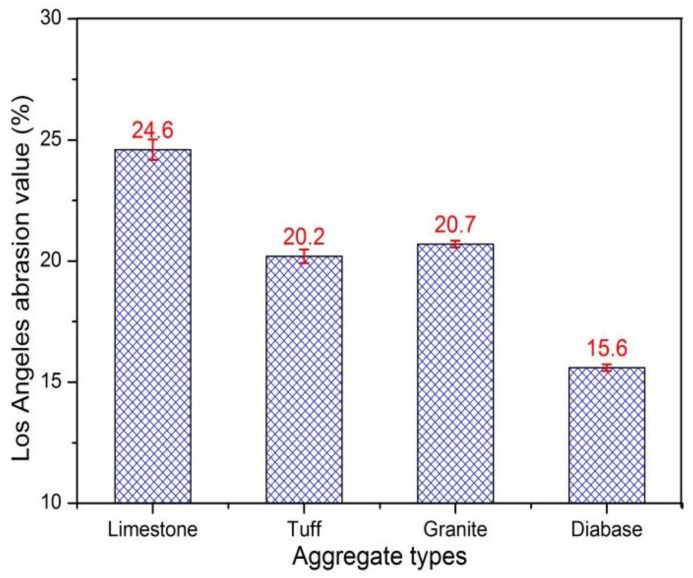
Abrasion values of different types of coarse aggregates.

Two sets of parallel tests for each type of aggregate were conducted, and the average results were shown in Figure 3. It can be seen from Figure 3 that the limestone aggregates had the largest abrasion value, followed by granite, tuff, and diabase. The diabase aggregate had the best abrasion resistance, indicating diabase aggregates had the highest ability to resist wheel impact and rub. Since the tuff aggregate was selected from iron ore tailings that contained lots of metal ions, resulting in its better abrasion resistance than granite aggregate. The highest abrasion value of limestone reflected the abrasion loss of limestone aggregates was largest, but detailed loss parts needed to be known from the morphological characteristic.

**Figure 4:**
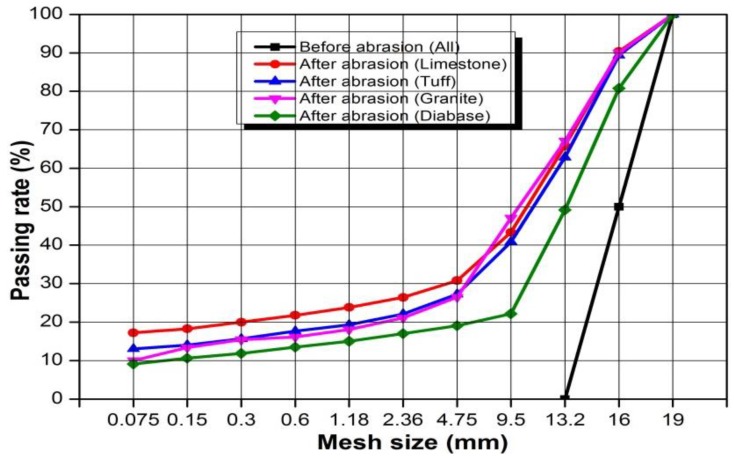
Gradation curves of aggregates before and after abrasion test.

The gradation curves of the aggregates before and after abrasion test were shown in Figure 4. In this study, aggregates of 13.2–16 mm and 16–19 mm were used in the Los Angeles abrasion test. Obviously, all the four types of aggregates are distributed in various sizes after abrasion and the majority is in the 4.75–19 mm. And the distribution results are consistent with the abrasion results. The diabase produced least fine aggregates after abrasion while limestone produced most fine aggregates after abrasion.

### 3.3. Morphological Characteristics Analysis

#### 3.3.1. Angularity

**Figure 5:**
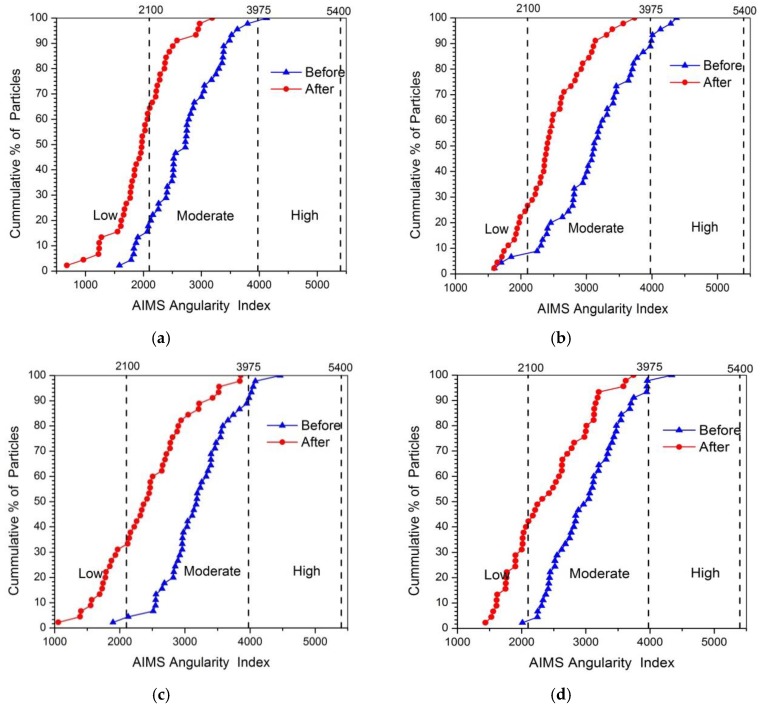
Percentage cumulative distribution of angularity before and after abrasion (**a**) Limestone; (**b**) Tuff; (**c**) Granite; (**d**) Diabase.

**Figure 6:**
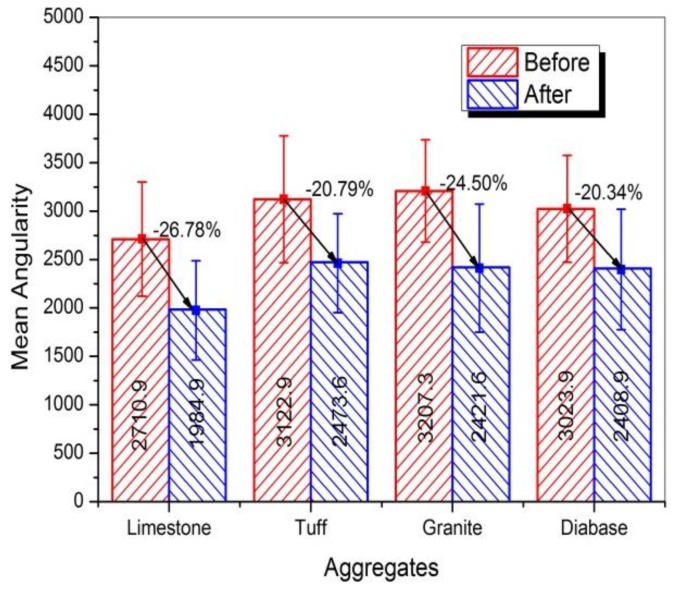
Mean angularity of coarse aggregates before and after abrasion test.

**Figure 7:**
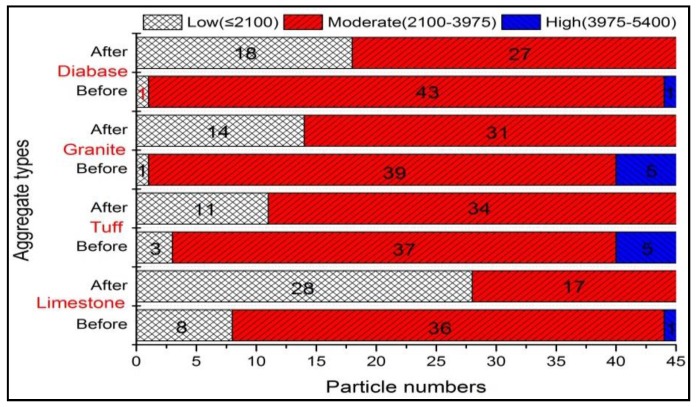
Angularity distribution of aggregates before and after abrasion test.

Figure 5 showed the plot of angularity for four kinds of coarse aggregates (i.e., limestone, tuff, granite, and diabase) before and after abrasion test. Obviously, the angularity value for each type of aggregate significantly reduced after abrasion, which was consistent with the results of Mahmoud’s research [23]. During the abrasion process, the angular areas of aggregates will rub against the steel ball first, causing the aggregate becoming less angular after abrasion. However, there was an obvious difference among the angularity distributions for various types of coarse aggregates, which might be caused by the differences in mineral compositions. The mean angularity of different aggregates was shown in Figure 6, and variation in angularity distribution of coarse aggregates can be further understood by comparing the number of particles in each angularity range (low, moderate and high), as plotted in Figure 7.

It can be seen from Figure 6 that the mean value of angularity for limestone aggregate was reduced by 26.78%, from 2710.9 to 1984.9, which was the largest. Conversely, the diabase aggregates had the smallest angularity reduction, followed by granite and tuff, which was consistent with the results of abrasion test. This reduction difference might be caused by the differences in the mineral compositions of coarse aggregates. During the abrasion process, the calcite in limestone aggregate was easy to abrade due to their low hardness, so the edges and corners of the aggregate were easily smoothed, resulting in a large abrasion value. The quartz and albite made diabase and granite have higher resistant to abrasion, and more albite and less calcite made tuff have better abrasion resistant comparing to calcite. Figure 7 showed that before abrasion test, there were a higher percentage of aggregates in moderate and high ranges of angularity and a lower percentage of aggregates in low range of angularity, indicating that abrasion test produced a high percentage of aggregates in low range of angularity. Such variations in individual ranges of angularity resulted in a different distribution in Figure 5.

Overall, the angularity of various coarse aggregates would be reduced after abrasion. However, due to the differences in the mineral composition of coarse aggregates, the angularity reduction for various types of aggregates was different, which was consistent with the results of abrasion test. The calcite in limestone aggregate was easy to abrade due to their low hardness, while the quartz and albite made diabase and granite have higher resistant to abrasion. Therefore, for pavements with high anti-skid requirement, the coarse aggregates with large angularity and more quartz and albite content should be preferred.

#### 3.3.2. Surface Texture

**Figure 8:**
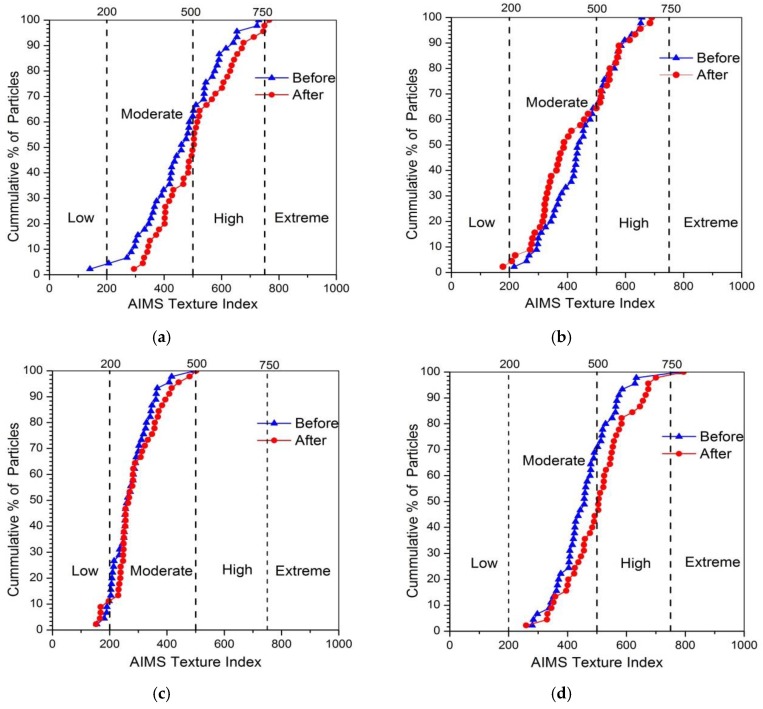
Percentage cumulative distribution of texture before and after abrasion. (**a**) Limestone; (**b**) Tuff; (**c**) Granite; (**d**) Diabase.

**Figure 9:**
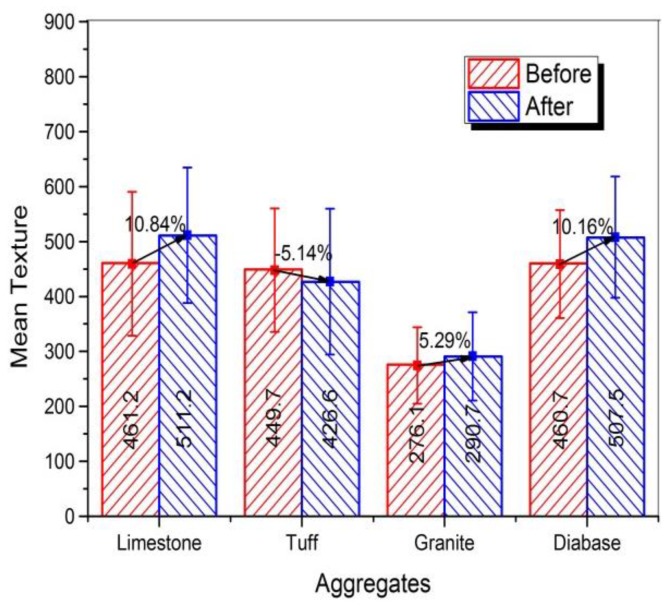
Mean texture of different coarse aggregates before and after abrasion test.

**Figure 10:**
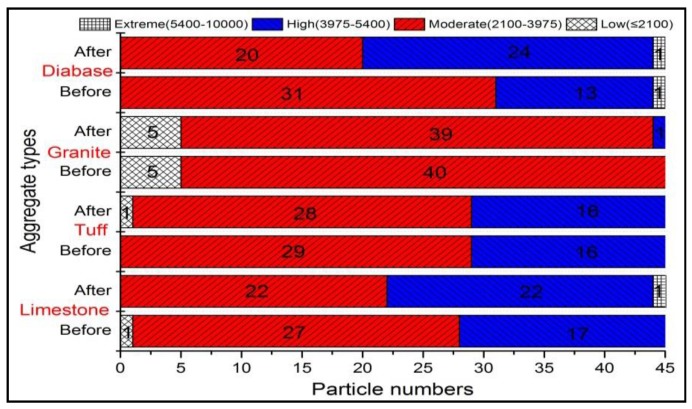
Texture distribution of different aggregates before and after abrasion test.

The plot of texture for four kinds of coarse aggregates before and after abrasion test was shown in Figure 8. Unlike the angularity, the surface texture of coarse aggregates did not reduce after abrasion, and even increased slightly for some types of aggregates. This result indicated that the abrasion test cannot be able to affect the microscopic surface texture of coarse aggregates. The slight increase of texture might be caused by the new breakage surface appeared in a small amount of aggregates during the abrasion process. The mean texture of different aggregates was shown in Figure 9, and variation in texture distribution of coarse aggregates was plotted in Figure 10.

Figure 9 showed that except for tuff aggregate, the mean value of surface texture for the other three aggregates increased slightly after abrasion. The mean value of texture for limestone increased by 10.84%, which was the largest. The reason might be that the low hardness of calcite in limestone caused the limestone easy to crush and produced more breakage surface. Since the tuff aggregate contained more metal ions and was hard to break, so it might be the reason for the reducing of texture after abrasion. Figure 10 showed that there was a large percentage in the moderate and high texture ranges of approximately 90% or higher for both before and after abrasion. Only few particles in the extreme range of texture were produced after abrasion because the crushing only occurred in a small amount of aggregates during the abrasion process.

In general, the texture value for different kinds of coarse aggregates didn’t reduce after abrasion test, indicating the abrasion cannot be able to affect the microscopic surface texture of coarse aggregates. The slight increase of texture might be caused by the new breakage surface appeared in a small amount of aggregates during the abrasion process. The calcite in limestone aggregates was easy to break because of its low hardness, causing the largest texture increase of limestone. So aggregates with less calcite content are recommended.

#### 3.3.3. Sphericity

**Figure 11:**
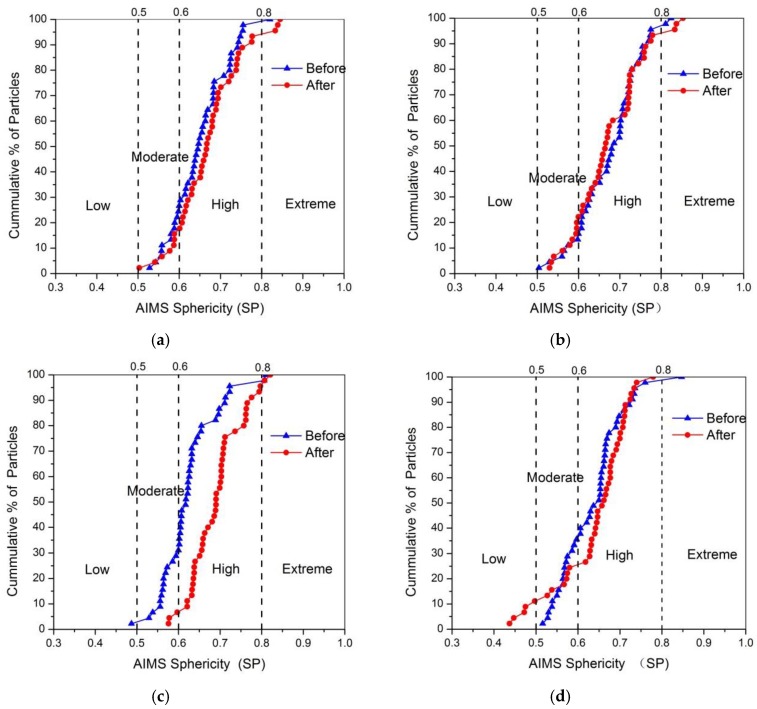
Percentage cumulative distribution of sphericity before and after abrasion (**a**) Limestone; (**b**) Tuff; (**c**) Granite; (**d**) Diabase.

**Figure 12:**
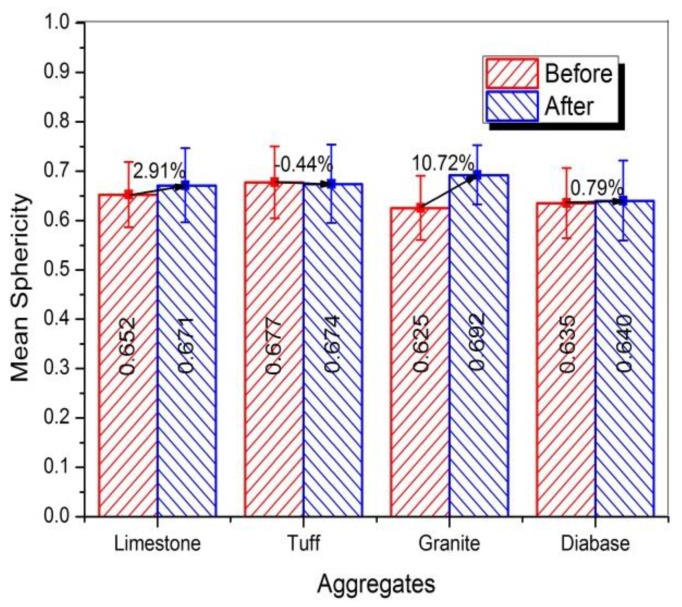
Mean sphericity of coarse aggregates before and after abrasion test.

**Figure 13:**
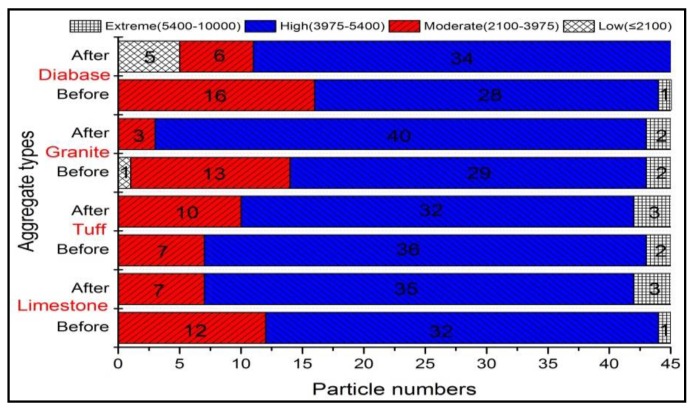
Sphericity distribution of aggregates before and after abrasion test.

Figure 11 showed the percentage of the cumulative distribution of sphericity for four kinds of aggregates before and after abrasion. It can be obviously seen that all the sphericity for four types of aggregates was slightly improved after abrasion, especially for the granite aggregates. The slight increase of sphericity was mainly caused by the smoothing of edges and corners of coarse aggregates. The breaking of flat or elongated particles was another reason for the increasing of sphericity. So the presence of more flat or elongated particles may be responsible for the greater improved sphericity of the granite aggregates, which would be confirmed by the next section. The mean sphericity value for different aggregates was shown in Figure 12, and variation in sphericity distribution of coarse aggregate was plotted in Figure 13.

Figure 12 showed except for granite aggregates, there was no significant difference for the mean value of sphericity before and after abrasion test. The mean sphericity of granite aggregates was increased by 10.72%, higher than that of other type aggregates, indicating there were more flat or elongated particles in the granite aggregates. The sphericity distribution in Figure 13 showed that some aggregates in low and moderate sphericity ranges transferred to high sphericity range after abrasion, whereas very few aggregates in high or moderate sphericity range transferred to low sphericity range. The reason was that the flat or elongated particles are easier to break comparing with the round particles.

In short, the sphericity value of coarse aggregates would be slightly increased after abrasion test due to the reduction of angularity. The sphericity of granite aggregates increased largest, which might be caused by the more flat or elongated particles in the granite aggregates. In addition, the larger sphericity increase of limestone aggregates might be due to the more calcite was contained in limestone aggregates. Since the aggregates with larger F and E ratio or more calcite content are easy to break, coarse aggregates with less flat or elongated particles and calcite content were recommended for heavy traffic road.

#### 3.3.4. F and E Ratio

**Figure 14:**
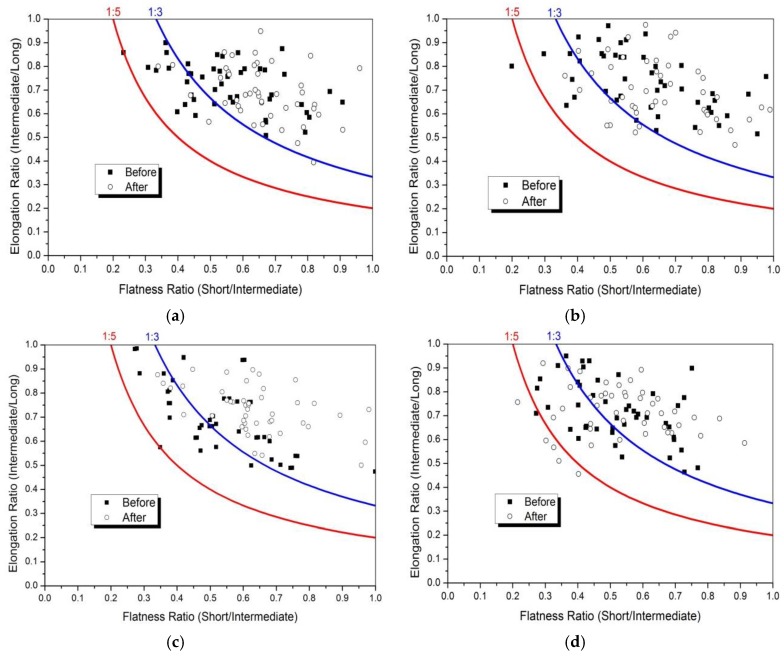
Percentage cumulative distribution of F and E before and after abrasion. (**a**) Limestone; (**b**) Tuff; (**c**) Granite; (**d**) Diabase.

**Figure 15:**
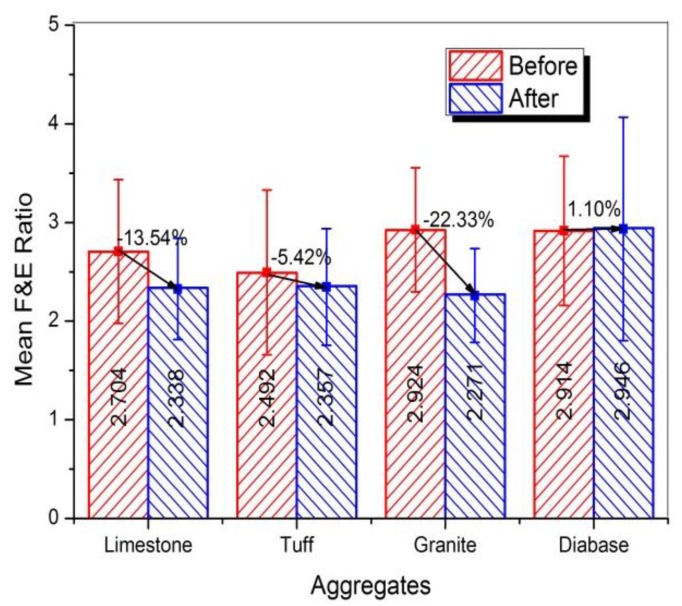
Mean F and E ratio of different aggregates before and after abrasion test.

**Figure 16:**
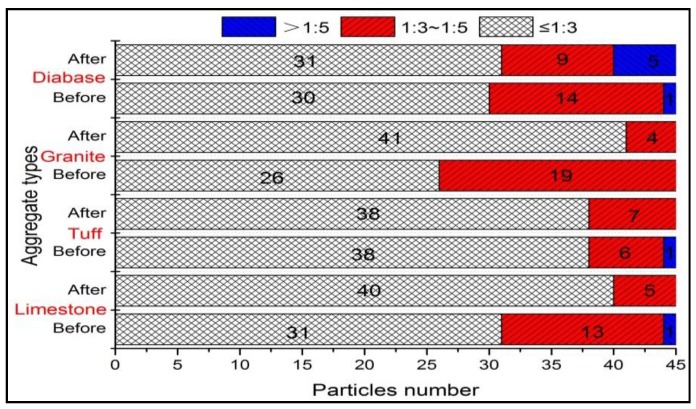
F and E distribution of different aggregates before and after abrasion test.

To compare the distribution of F and E ratio before and after abrasion, flatness (*L_S_/L_I_* index) versus elongation (*L_I_*/*L_L_* index) data for four kinds of aggregates were plotted in Figure 14. Most aggregates distributed in the range of an aspect ratio of 1:3, whereas very few aggregates were in the range of an aspect ratio of 1:5. Obvious, the abrasion test produced a lower percentage of coarse aggregates with an aspect ratio of 1:3 to 1:5. The reason was that coarse aggregate particles with excessively high F and E ratio had high probability of breakage under abrasion. The mean F and E ratio value for different aggregates was shown in Figure 15, and variation in F and E ratio distribution of coarse aggregate was plotted in Figure 16.

Obviously, Figure 15 showed that the granite aggregates before abrasion had the highest F and E ratio, which can be used to confirm the greater improved sphericity of the granite aggregates after abrasion. The largest reduction of F and E ratio indicated that the flat or elongated particles were easy to break and should be controlled. Compared to other two types of aggregates, limestone aggregates had the larger reduction of F and E ratio, which might be caused by its high content of calcite. Figure 16 showed that most coarse aggregates with an aspect ratio of 1:3 to 1:5 transferred to the range of an aspect ratio of 1:3, which was reflected in the different distribution in Figure 14.

In summary, consistent with the results of sphericity, aggregates with excessively high F and E particles or more calcite content were easy to break and then change the shape characteristics, which were easy to cause rutting and were harmful to pavement. So the quantity of excessively high F and E particles should be controlled and aggregates with less calcite content are preferred.

## 4. Conclusions

In this study, the Los Angeles abrasion test was used to simulate the similar force exerted by wheel on coarse aggregates and the effect of abrasion test on morphologies of different coarse aggregates was investigated. Four types of coarse aggregates, including limestone, tuff, granite, and diabase, were selected. The morphological characteristics of different coarse aggregates before and after the abrasion test were measured and analyzed. Based on detailed results and discussion the following conclusions can be drawn:

(1) The different mineral compositions resulted in the different abrasion resistant of coarse aggregates. During the abrasion process, the calcite in limestone aggregate was easy to abrasion due to their low hardness. The quartz and albite made diabase and granite have higher resistant to abrasion, and the more albite and less calcite made tuff have better abrasion resistant comparing with calcite.

(2) The angularity value for each type of aggregate significantly reduced after abrasion. However, the angularity reduction for various aggregates was obviously different. Consistent with the abrasion value results, the limestone aggregates had the largest angularity reduction, followed by granite, tuff, and diabase, indicating the angularity reduction was the main component of abrasion loss. Unlike the angularity, there was no significant different between the surface texture of coarse aggregates before and after abrasion, indicating the abrasion test cannot be able to affect the microscopic surface texture of coarse aggregates. Therefore, for pavements with high anti-skid requirement, the coarse aggregates with large angularity and low abrasion value should be preferred.

(3) The sphericity and F and E ratio for four types of aggregates was both slightly improved after abrasion due to the smoothing of edges and corners of coarse aggregates, which can reflect the angularity reduction was the main component of abrasion loss. Consistent with the results of sphericity, F and E ratio results showed that aggregates with excessively high F and E particles were easy to break, which was harmful to the stability of pavement. So the quantity of excessively high F and E particles should be controlled.

The morphology changes of different aggregates after abrasion were identified in this study, which has practical guidance for selecting the right aggregates and improving the pavement performance of asphalt mixtures. Because of the limited scope, the current study only considered four kinds of aggregates and one particle size combination for abrasion. Therefore, it is recommended that future work be carried out for other types of aggregates. Further, aggregates with different gradation combinations can be used for abrasion tests. In addition, the performance of asphalt mixtures may be studied to better capture the role of changes in morphologies.
